# Chinese Altay sheep adapt to seasonal fluctuations in forage nutrients by changing physiological status and rumen microbial community structure

**DOI:** 10.3389/fmicb.2025.1720729

**Published:** 2025-12-16

**Authors:** Mingyu Ma, Peng Zhang, Zhenzi Xu, Shu Li, Sijin Li, Qicheng Lu, Wenju Zhang, Yuanyaun Li

**Affiliations:** College of Animal Science and Technology, Shihezi University, Shihezi, China

**Keywords:** grazing, Altay sheep, blood characteristics, rumen fermentation, rumen microbiota

## Abstract

**Introduction:**

As the primary nutritional source for grazing livestock, natural forage directly influences changes in physiological status and rumen microbial community structure owing to seasonal variations in its nutritional composition. However, the physiological and rumen microbial community adaptations of Chinese Altay sheep in response to seasonal variations in forage nutrition remain unclear.

**Methods:**

Therefore, this study systematically evaluated the effects of seasonal dynamics in forage nutrition on growth performance, nutrient digestibility, blood parameters, rumen fermentation characteristics, and rumen microbiota in grazing Chinese Altay sheep. A total of 18 grazing Altay growing ewes with similar body weight, age, and good health condition were selected for the experiment. During four typical seasons (spring green-up, summer lush, autumn withering, and winter dormancy), Altay sheep were administered C_32_ slow-release capsules, with concurrent collection of forage, feces, blood, and rumen content samples.

**Results:**

The results indicated that the nutrient content of forages was abundant in summer and autumn, leading to high feed intake by the Altay sheep. This enabled the animals to promote weight gain through enhanced nutrient digestibility and rumen fermentation efficiency. Blood biochemical parameters of the Altay sheep varied in response to seasonal changes and forage nutrient availability. The results demonstrated that during summer, grazing Altay sheep exhibited a decline in antioxidant capacity, as well as reduced concentrations of Immunoglobulin A (IgA), Immunoglobulin G (IgG), and Immunoglobulin M (IgM). In autumn, significant enrichment of Bacillota and *NK4A214_group* was observed in the rumen of grazing sheep, which contributed to enhanced nutrient acquisition and thereby promoted fat deposition. In winter, *Rikenellaceae_RC9_gut_group* and *Prevotellaceae_UCG-003* were significantly enriched in the rumen of grazing sheep, facilitating fiber decomposition and the production of volatile fatty acids (VFAs).

**Discussion and conclusion:**

This microbial adaptation helped the animals cope with nutrient scarcity by meeting their nutritional requirements. In conclusion, Altay sheep adapted to seasonal forage and environmental fluctuations through coordinated changes in feed intake, blood parameters, and rumen microbiota, thereby ensuring their survival.

## 1 Introduction

Grazing represents a significant mode of animal husbandry, particularly in resource-limited regions such as arid, semi-arid, and alpine areas. It not only provides a vital livelihood for pastoralists but also enables efficient utilization of natural grassland resources, maintains ecological balance, and helps address environmental challenges associated with climate change. As the primary nutritional source for grazing livestock, forage nutritional composition directly affects the growth performance and health status of animals. However, the fluctuation in forage nutrient supply caused by seasonal variation represents a key bottleneck constraining the sustainable development of grazing animal production. Research has shown that the crude protein content of forage is significantly higher during the peak growing season compared to the dormant period, while the fiber content exhibits the opposite seasonal pattern (Firkins and Yu, 2015). These seasonal nutrient fluctuations influence not only the physiological characteristics of livestock but also their rumen fermentation and microbial community structure (Liu et al., 2020).

The rumen, a core organ of the ruminant digestive system, hosts a diverse microbial community, comprising bacteria, archaea, ciliates, fungi, and viruses (Li and Guan, 2017). These symbiotic microorganisms ferment complex plant fibers and polysaccharides, producing essential volatile fatty acids, vitamins, and microbial proteins that support host growth and physiological maintenance (Crawford et al., 2009). The rumen enhances host nutrition and energy metabolism not only by promoting feed energy absorption and storage (Wahrmund et al., 2012), but also via dynamic thermal regulation through constant heat exchange with the environment. Evidence indicates that environmental temperature can significantly influence rumen temperature. Under colder conditions, this thermal interaction may lower body temperature and compromise the immunity and health of ruminants (Wang S. et al., 2023). Studies have shown that during cold seasons, Tibetan sheep adjust their physiological and nutritional adaptation strategies, along with the structure and function of the rumen microbial community, to cope with seasonal shifts characterized by reduced feed availability and quality (Cui et al., 2023).

The Altay sheep is a distinctive breed indigenous to the Altay region of Xinjiang, China. It is characterized by rough feed tolerance, cold resistance, disease resistance, and efficient nutrient utilization, exhibiting considerable phenotypic plasticity in response to seasonal environmental variations and fluctuations in forage resources (Zhou et al., 2020). However, its physiological adaptation mechanism under seasonal grazing conditions is still unclear. Therefore, this study aims to investigate the effects of seasonal fluctuations in forage nutrition on the growth performance, nutrient digestibility, blood biochemical parameters, immune function, antioxidant capacity, and rumen microbiota composition of grazing Altay sheep. The findings are expected to provide a theoretical foundation for understanding ruminant adaptation to grazing environments and to offer a scientific basis for optimizing seasonal grazing management strategies, thereby enhancing the productivity of grassland-based animal husbandry.

## 2 Materials and methods

### 2.1 Animals, experimental design

The study was conducted between March 2024 and February 2025 in Qiganjiedie Township, Fuhai County, located in the Altay region of Xinjiang, China. The study area experiences a temperate continental climate, characterized by a mean annual temperature of 3.9 °C. The forage green-up period typically begins in March, with senescence occurring by September. The dominant pasture species comprised *Stipa caucasica Schmalh*, *Festuca valesiaca subsp., Artemisia frigida Willd*, and *Cleistogenes squarrosa*. The study encompassed four distinct seasonal periods with sampling conducted in: May 2025 (The study encompassed four distinct seasonal periods: the spring grass green-up period), July 2024 (summer vigorous growth period), October 2024 (autumn grass withering period), and December 2024 (winter dry grass period). During the experiment, 18 healthy grazing Altay ewes with similar body weight and age (35.2 ± 1.05 kg; 6 months old) were randomly selected from a pasture in Altay, Xinjiang. Each experimental period spanned 13 days, comprising an 8-day adaptation phase followed by a 5-day formal trial period. Prior to the commencement of the study, all experimental sheep were fitted with ear tags for identification. During the trial, the sheep were released for daily grazing at 10:00 and returned to the barn at 19:30. Each morning prior to grazing, saturated alkane C_32_ slow-release pellets were administered to the sheep using a dosing gun. The release rate of the saturated alkane C_32_ pellets was 48.5 mg/d. Both the C_32_ pellets and their specified release rate were provided by the Animal Nutrition Laboratory of Tarim University. The saturated alkane technique was employed to estimate the daily forage intake of the grazing sheep. The management of the experimental sheep was consistent with standard pasture practices, and no supplementary feeding was provided throughout the study duration.

### 2.2 Sample processing

On the day following the administration of alkane slow-release pellets, forage samples were collected using the simulated grazing method. During the pre-trial period, sheep were grazed daily. The species, plant parts, and height of the forage consumed by the sheep were observed. Forage samples were obtained using scissors following a diagonal sampling pattern, with unpalatable plants excluded. Samples from each area were placed in separate cloth bags, labeled, then oven-dried at 65 °C and ground to pass through a 40-mesh sieve for subsequent analysis. During the formal trial period, fecal samples were collected twice daily via rectal sampling before morning grazing and upon return from grazing. Feces from each sheep were thoroughly mixed, placed in sealed bags, and stored at −20 °C. Subsequently, 10% of the total collected feces was randomly selected, weighed, and dried for analysis. Dry matter (DM) content in forage and fecal samples was determined according to AOAC Official Method 925.04. The crude protein (CP) content was measured by the Kjeldahl method using a semi-automatic nitrogen analyzer (Kjeltec-8200, Denmark) following AOAC Official Method 990.03. Ether extract (EE) content was analyzed with a fully automatic fat analyzer (XT15i, USA) as per AOAC Official Method 2003.05. The crude ash (Ash) content was determined using a muffle furnace (SX2-4-10, China) according to AOAC Official Method 942.05. The neutral detergent fiber (NDF) and acid detergent fiber (ADF) contents were determined using the method described by Soest et al. (1991). Sheep were weighed on the first and last days of each trial period to calculate average daily gain (ADG). The acid-insoluble ash (AIA) content in the forage and fecal samples was determined according to the method described by Soest et al. (1991) and was used as an internal marker for the calculation of apparent digestibility, as per the standard method. The daily dry matter intake (DMI) was estimated using the saturated alkane technique (Mayes et al., 1986).

On the day before the end of each trial period, blood was collected via the jugular vein from the Altay sheep prior to their release for grazing, and placed in tubes containing an anticoagulant (sodium heparin). The tubes were then centrifuged at 3000 × *g* for 15 min, and the plasma was collected and stored at −20 °C for testing. Plasma total protein (TP), albumin (ALB), globulin (GLB), blood urea nitrogen (BUN), alkaline phosphatase (ALP), total cholesterol (TC), triglycerides (TG), aspartate transaminase (AST), alanine transaminase (ALT), and lactate dehydrogenase (LD) levels or activity were measured using a Roche Cobas 8000 C702 fully automated biochemical analyser. Antioxidant and immune indicators in blood were measured using enzyme-linked immunosorbent assay (ELISA) kits purchased from Suzhou Greetech Biotechnology Co., Ltd. Concentrations of growth hormone (GH), insulin-like growth factor-1 (IGF-1), triiodothyronine (T3), and thyroxine (T4) in plasma were assessed using specific ELISA kits (Jiangsu Jingmei Biotechnology Co., Ltd., China). All assays were performed in strict accordance with the manufacturers’ protocols.

On the final three consecutive days of each experimental period, rumen fluid was collected from Altay sheep 2 h prior to morning release for grazing, using an oral rumen fluid sampler. This single timepoint was selected to standardize sample collection across all seasons and animals, capturing a representative baseline state of the rumen environment before the intake of fresh forage, thereby enabling a consistent comparison of seasonal effects. The collected rumen fluid was sequentially filtered through four layers of cheesecloth followed by a 60-μm nylon mesh. The pH of the filtrate was immediately determined using a calibrated portable pH meter. Following pH measurement, samples were immediately aliquoted into cryogenic vials, snap-frozen in liquid nitrogen, transported to the laboratory, and stored at −80 °C until analysis. Volatile fatty acid (VFA) concentrations were determined by gas chromatography (Agilent 7890B, USA) using the internal standard method. Ammonia nitrogen (NH₃-N) concentration was analyzed colorimetrically using a spectrophotometer (UV-1700, Shimadzu, Japan).

### 2.3 Rumen bacterial 16S rDNA sequence determination and bioinformatics analysis

Total soil microbial DNA was extracted using the E.Z.N.A.® Soil DNA Kit. The quality, concentration, and purity of the DNA were assessed by agarose gel electrophoresis and a NanoDrop2000 spectrophotometer. Using the extracted DNA as a template, the V3-V4 hypervariable region of the bacterial 16S rRNA gene was amplified with barcoded primers 338F and 806R, followed by PCR purification for the construction of sequencing libraries. Sequencing was performed on an Illumina NextSeq 2000 platform. Raw reads were quality-filtered using fastp and assembled using FLASH. Operational Taxonomic Units (OTUs) were clustered with 97% sequence similarity using UPARSE, and chimeric sequences were removed. After discarding sequences affiliated with chloroplasts and mitochondria, all samples were rarefied to 20,000 sequences per sample to ensure a coverage of 99.09%. Taxonomic annotation was performed using the RDP classifier against the SILVA database (v138). Data analysis, including taxonomic classification at different levels, beta diversity, alpha diversity, and analysis based on the rRNA database, was conducted using software and databases such as QIIME 1.91 and SILVA 138. These specific analytical processes were carried out on the Majorbio Cloud Platform. Raw sequences were submitted to the NCBI the Biotechnology Information Sequence Read Archive (accession number PRJNA1308996).

### 2.4 Statistical analysis

All statistical analyses were performed using IBM SPSS Statistics (Version 25.0, IBM Corp., USA). Prior to analysis, all data were tested for normality using the Shapiro–Wilk test and for homogeneity of variances using Levene’s test. For data meeting the assumptions of parametric tests, one-way analysis of variance (ANOVA) was used for comparisons among groups. If the ANOVA results indicated significant differences, a Tukey test was applied for post-hoc comparisons. For data that did not meet the assumptions of parametric tests, non-parametric tests (such as the Kruskal–Wallis H test) were employed for analysis. The results are presented in tables, with data shown as the mean and the standard error of the mean (SEM) listed separately. Statistical significance was set at *p* < 0.05.

## 3 Results

### 3.1 The dynamic changes of nutritional components of forage grass with seasons

As shown in Table 1, there are differences in the nutritional components of natural forage across different seasons. CP content in summer forage was significantly higher than in other seasons, and CP content in autumn forage was significantly higher than in winter and spring (*p* < 0.05). EE content in autumn forage was significantly higher than in other seasons (*p* < 0.05). ADF, NDF, and Ash contents in winter forage were the highest among the four seasons, ADF content in summer forage was the lowest, and NDF content in autumn forage was the lowest (*p* < 0.05).

**Table 1:** Effect of grazing season on nutritional components of pasture grass (%).

Items	Season				SEM	*p*-value
	Spring	Summer	Autumn	Winter		
CP	7.43^c^	10.73^a^	9.59^b^	7.18^c^	0.472	<0.01
EE	4.74^b^	6.03^b^	7.57^a^	4.91^b^	0.371	<0.01
NDF	62.43^b^	59.02^b^	54.95^c^	68.44^a^	1.557	<0.01
ADF	27.20^b^	21.90^c^	26.28^b^	33.30^a^	1.29	<0.01
Ash	8.60^b^	7.85^b^	7.36^b^	10.45^a^	0.393	<0.01

There are significant differences with different letters (a, b, c) on the shoulder of the same index (*p* < 0.05). CP, crude protein; EE, ether extract; NDF, neutral detergent fiber; ADF, acid detergent fiber; Ash, crude ash.

### 3.2 The effect of grazing season on average daily weight gain, feed intake, and apparent nutrient digestibility in Altay sheep

**Figure 1:**
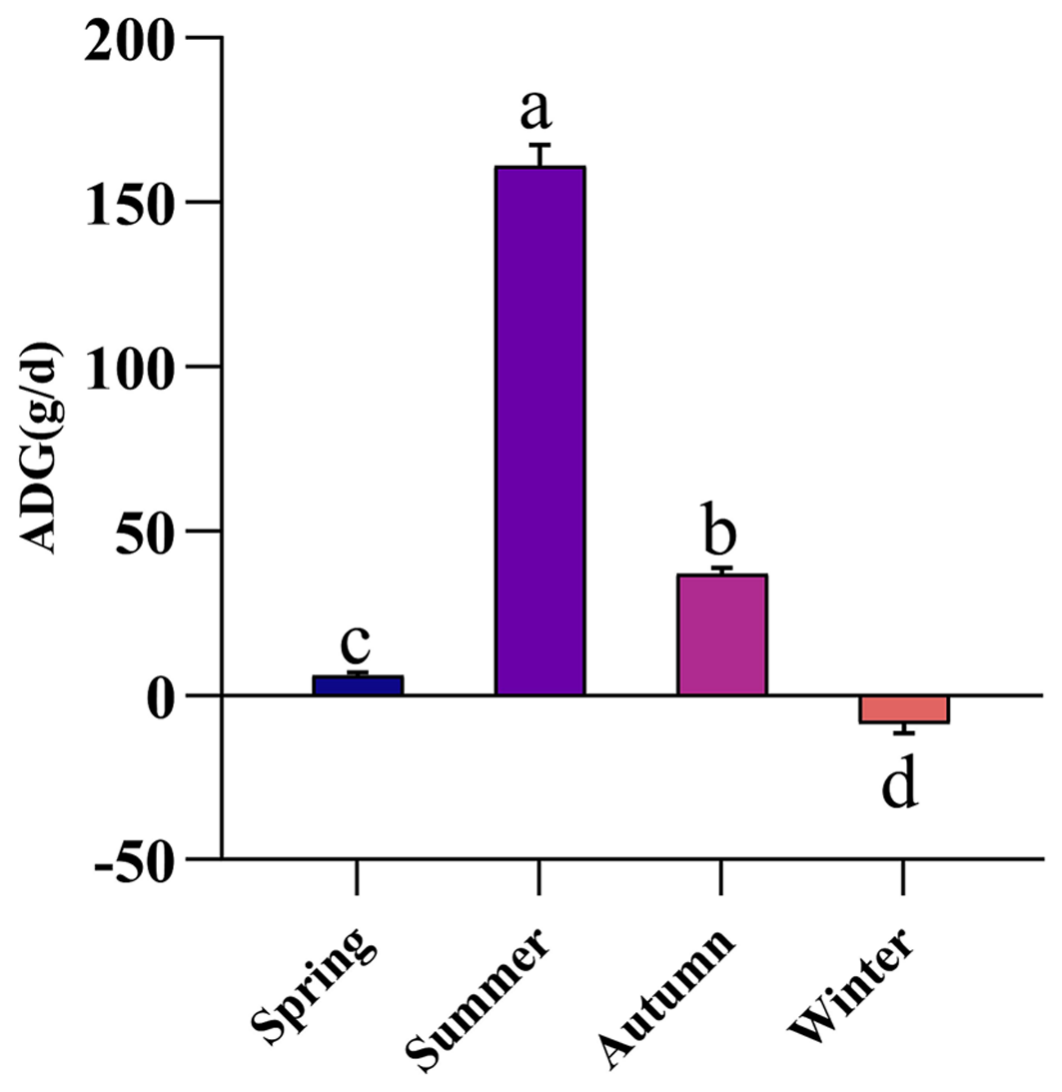
The effect of different seasons on average daily gain of grazing sheep. ADG, average daily gain. The same letter or no letter indicates no significant difference (*p* > 0.05), while different letters indicate a significant difference (*p* < 0.05). The same applies to the figure below.

As presented in Figure 1, the average daily gain (ADG) of grazing Altay sheep during spring, summer, autumn, and winter was 6.03, 161.03, 36.9, and −8.7 g/d, respectively. Significant differences in ADG were observed among seasons (*p* < 0.05). Specifically, the ADG in summer was significantly higher than that in other seasons, while winter was characterized by a marked weight stasis.

As shown in Table 2, there were significant differences in the seasonal dry matter intake (DMI) of grazing Altay sheep. The autumn DMI was significantly higher than that of other seasons (*p* < 0.05), followed by summer, with the lowest DMI in winter. In summer, the digestibility of CP, EE, NDF, ADF, and dry matter (DM) in pasture forage was significantly higher than that in winter (*p* < 0.05).

**Table 2:** The effect of grazing season on nutrient apparent digestibility in sheep.

Items	Season				SEM	*p*-value
	Spring	Summer	Autumn	Winter		
DMI (g/d)	892.25^c^	1149.22^b^	1332.57^a^	724.90^d^	70.624	<0.01
CP (%)	55.45^b^	62.87^a^	58.79^ab^	48.73^c^	1.730	<0.01
EE (%)	60.46^ab^	67.46^a^	64.25^ab^	58.55^b^	1.279	0.03
NDF (%)	43.35^b^	52.54^a^	45.31^b^	36.78^c^	1.826	<0.01
ADF (%)	26.35^b^	34.39^a^	30.73^ab^	24.32^c^	1.421	0.02
DM (%)	60.38^ab^	66.70^a^	62.35^ab^	57.27^b^	1.295	0.04

There are significant differences with different letters (a, b, c, d) on the shoulder of the same index (*p* < 0.05). DMI, dry matter intake; CP, crude protein; EE, ether extract; NDF, neutral detergent fiber; ADF, acid detergent fiber; DM, dry matter.

### 3.3 The effect of grazing season on blood biochemical indicators of Altay sheep

The effects of grazing season on blood biochemical parameters of Altay sheep are presented in Table 3. The ALT concentration in autumn was significantly higher than that in winter (*p* < 0.05), while no significant differences were observed among spring, summer, and winter. During summer and autumn, TP was significantly higher than that in winter (*p* < 0.05), with ALB and AST being significantly elevated compared to spring and winter (*p* < 0.05). Winter exhibited significantly lower GLB and TG concentrations compared to other seasons (*p* < 0.05). Both ALP and BUN in spring and autumn were significantly higher than those in summer and winter (*p* < 0.05), while TC was significantly higher than that in winter (*p* < 0.05). Additionally, LD in spring was significantly higher than in autumn and winter (*p* < 0.05). The concentrations of GH, *T3*, and *T4* in spring were significantly higher than those in other seasons (*p* < 0.05). IGF-1 demonstrated an initial decrease followed by an increase across seasons, with its peak value in spring being significantly higher than in other seasons (*p* < 0.05).

**Table 3:** The effect of different seasons on blood biochemical parameters of grazing sheep.

Items	Season				SEM	*p*-value
	Spring	Summer	Autumn	Winter		
ALT (U/L)	15.79^ab^	18.92^ab^	20.93^a^	13.61^b^	0.816	<0.01
TP (g/L)	59.40^ab^	65.38^a^	66.96^a^	53.46^b^	1.292	<0.01
ALB (g/L)	25.10^b^	30.47^a^	31.11^a^	24.49^b^	0.617	<0.01
GLB (g/L)	34.32^a^	35.41^a^	35.86^a^	28.97^b^	0.847	<0.01
AST (U/L)	88.85^b^	135.93^a^	115.82^a^	72.28^b^	4.828	<0.01
ALP (U/L)	165.9^a^	99.60^b^	231.62^a^	90.70^b^	13.659	<0.01
BUN (mmol/L)	8.15^a^	5.18^b^	7.82^a^	3.54^c^	0.330	<0.01
TG (mmol/L)	0.38^a^	0.41^a^	0.45^a^	0.16^b^	0.028	<0.01
TC (mmol/L)	1.70^a^	1.55^ab^	1.90^a^	1.29^b^	0.065	<0.01
LD (mmol/L)	435.40^a^	643.92^ab^	555.90^bc^	375.80^c^	23.895	<0.01
GH (ng/mL)	22.693^a^	11.04^c^	9.92^c^	19.13^b^	0.923	<0.01
IGF-1 (ng/mL)	390.13^a^	224.91^c^	181.78^d^	343.53^b^	14.734	<0.01
T3 (nmol/L)	8.69^a^	6.68^b^	6.79^b^	7.67^ab^	0.230	<0.01
T4 (nmol/L)	351.15^a^	167.84^c^	182.63^c^	290.29^b^	13.411	<0.01

There are significant differences with different letters (a, b, c, d) on the shoulder of the same index (*p* < 0.05). ALT, alanine aminotransferase; TP, total protein; ALB, albumin; GLB, globulin; AST, aspartate aminotransferase; ALP, alkaline phosphatase, BUN, blood urea nitrogen; TG, triglyceride; TC, total cholestero; LD, lactate dehydrogenase; GH, growth hormone; IGF-1, insulin-like growth factor-1; T3, triiodothyronine; T4, thyroxine.

### 3.4 The effect of grazing season on the antioxidant capacity of Altay sheep

**Figure 2:**
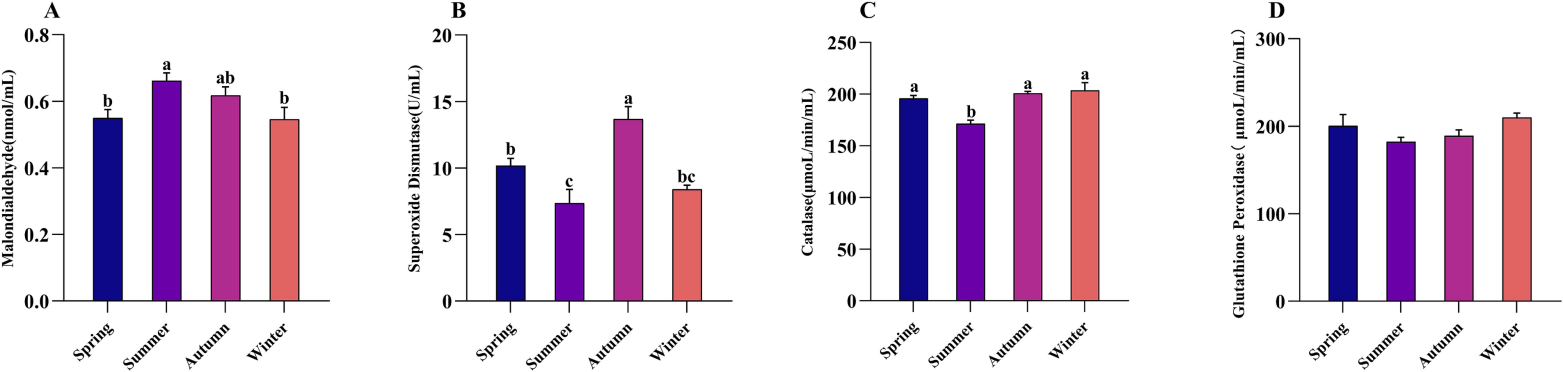
The effect of grazing season on the antioxidant capacity of Altay sheep. **(A)** Malondialdehyde; **(B)** Superoxide dismutase; **(C)** Catalase; **(D)** Glutathione peroxidase. The same letter or no letter indicates no significant difference (*p* > 0.05), while different letters indicate a significant difference (*p* < 0.05). The same applies to the figure below.

The effects of grazing season on the antioxidant capacity of Altay sheep are shown in Figure 2. The results indicated that the serum MDA concentration in summer was significantly higher than that in winter and spring (*p* < 0.05). No significant differences were observed in GSH-Px activity among seasons (*p* > 0.05). The superoxide dismutase (SOD) activity in autumn was significantly higher than that in other seasons, and the SOD activity in spring was significantly higher than that in summer (*p* < 0.05). Additionally, the CAT activity in summer was significantly lower than that in other seasons (*p* < 0.05).

### 3.5 The effect of grazing season on inflammatory factors and immune performance in Altay sheep

**Figure 3:**
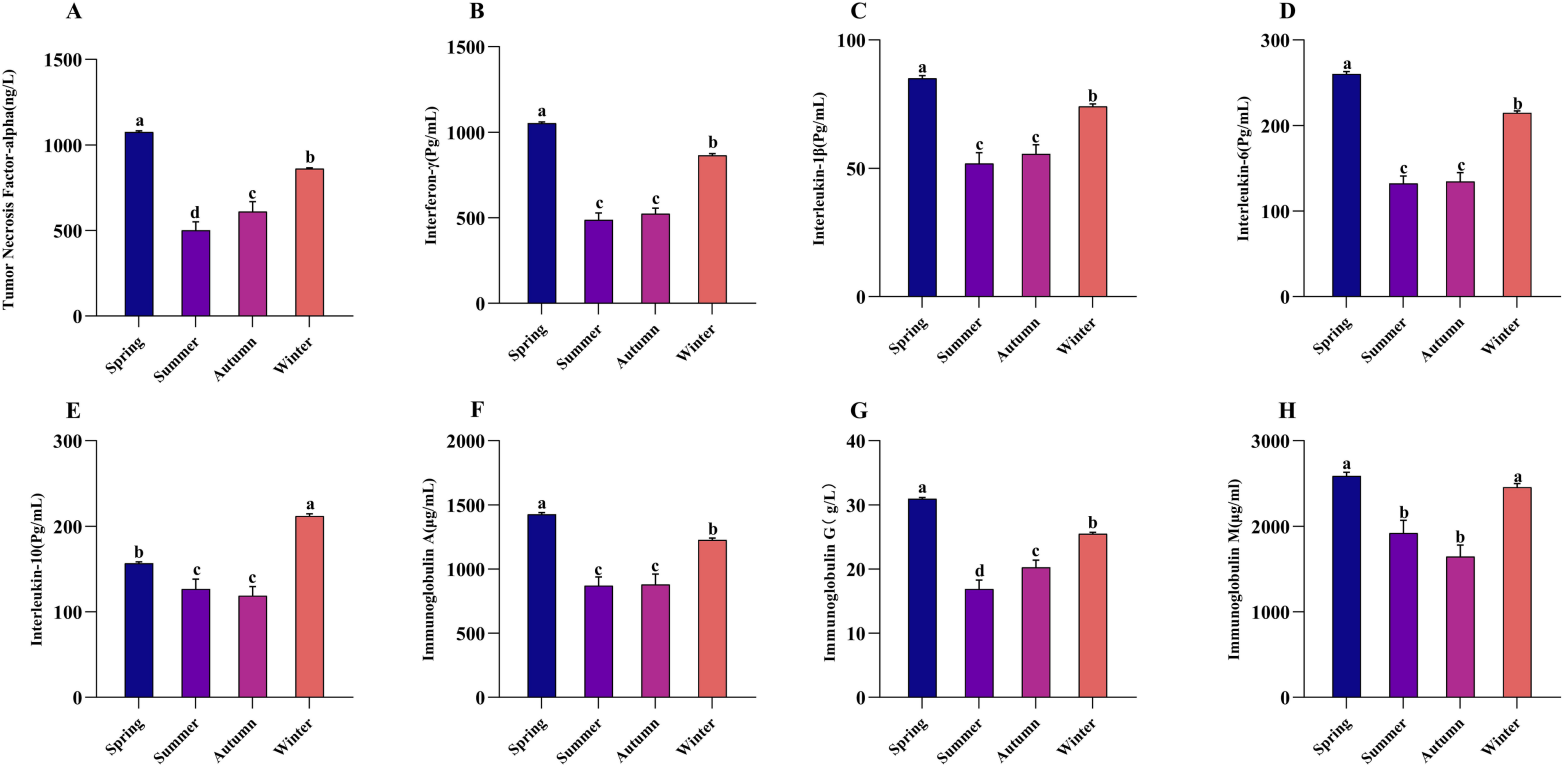
The effect of grazing season on inflammatory factors and immune performance in Altay sheep. Each bar represens the mean ± SEM. **(A)** Tumor necrosis factor-alpha; **(B)** Interferon-*γ*; **(C)** Interleukin-1β; **(D)** Interleukin-6; **(E)** Interleukin-10; **(F)** Immunoglobulin A; **(G)** Immunoglobulin G; **(H)** Immunoglobulin M. The same letter or no letter indicates no significant difference (*p* > 0.05), while different letters indicate a significant difference (*p* < 0.05). The same applies to the figure below.

The effects of grazing season on the immune performance of Altay sheep are presented in Figure 3. In spring, the serum concentrations of IFN-γ, IL-1β, IL-6, and IgA were significantly higher than those in other seasons (*p* < 0.05). Additionally, the concentrations of IFN-γ, IL-1β, IgA, and IL-6 in winter were significantly higher than those in summer and autumn (*p* < 0.05). The interleukin-10 (IL-10) concentration in winter was significantly higher than in other seasons (*p* < 0.05), with the lowest value observed in spring. The concentrations of immunoglobulin G (IgG) and tumor necrosis factor-*α* (TNF-α) demonstrated an initial decrease followed by an increase across seasons, reaching their peak values in spring that were significantly higher than those in other seasons (*p* < 0.05). The immunoglobulin M (IgM) concentration in spring and winter was significantly higher than that in summer and autumn (*p* < 0.05).

### 3.6 The effect of grazing season on rumen fermentation parameters in Altay sheep

The effect of grazing season on rumen fermentation parameters in Altay sheep is shown in Table 4. The pH of rumen fluid in Altay sheep grazing in summer was significantly lower than in other seasons (*p* < 0.05). The concentration of Ammonia Nitrogen (NH_3_-N) in rumen fluid was significantly higher in summer and autumn than in spring and winter (*p* < 0.05). The concentrations of acetic acid, propionic acid, and total volatile fatty acids in the rumen of sheep grazing in spring and summer were significantly higher than those in autumn and winter (*p* < 0.05); additionally, the concentrations of butyric acid, isobutyric acid, and isovaleric acid in the rumen of sheep grazing in winter were significantly lower than those in other seasons (*p* < 0.05). The concentration of valeric acid in the rumen of summer-grazed sheep was significantly higher than in other seasons, with the lowest concentration observed in winter (*p* < 0.05). The highest concentration of isobutyric acid was found in the rumen of spring-grazed sheep (*p* < 0.05). The acetic acid to propionic acid (A:P) ratio showed a trend of first decreasing and then increasing from spring to winter.

**Table 4:** The effect of different seasons on rumen fermentation parameters in grazing sheep.

Items	Season				SEM	*p*-value
	Spring	Summer	Autumn	Winter		
PH	7.38^a^	7.06^b^	7.31^a^	7.29^a^	0.301	0.01
NH_3_-N (mmol/L)	87.92^b^	178.21^a^	150.31^a^	113.40^b^	7.202	<0.01
Acetic acid (mmol/L)	296.02^a^	259.40^a^	143.00^b^	101.41^c^	14.162	<0.01
Propionic acid (mmol/L)	63.99^a^	62.07^a^	49.21^b^	14.31^c^	3.238	<0.01
Butyrate (mmol/L)	40.12^a^	42.64^a^	37.88^a^	6.48^b^	2.301	<0.01
Valerate (mmol/L)	0.95^b^	1.49^a^	0.95^b^	0.14^c^	0.078	<0.01
Isobutyric acid (mmol/L)	1.17^a^	0.93^b^	1.02^ab^	0.21^c^	0.060	<0.01
Isovalerate (mmol/L)	1.08^a^	0.89^b^	1.11^a^	0.20^c^	0.060	<0.01
Acetate/propionate	4.79^b^	4.14^c^	2.90^d^	6.96^a^	0.233	<0.01
Totalvolatilefatty acid (mmol/L)	403.45^a^	367.42^a^	233.57^b^	122.75^c^	19.145	<0.01

There are significant differences with different letters (a, b, c) on the shoulder of the same index (*p* < 0.05). NH_3_-N, Ammonia Nitrogen.

### 3.7 The effect of grazing season on the rumen microbiota of Altay sheep

**Figure 4:**
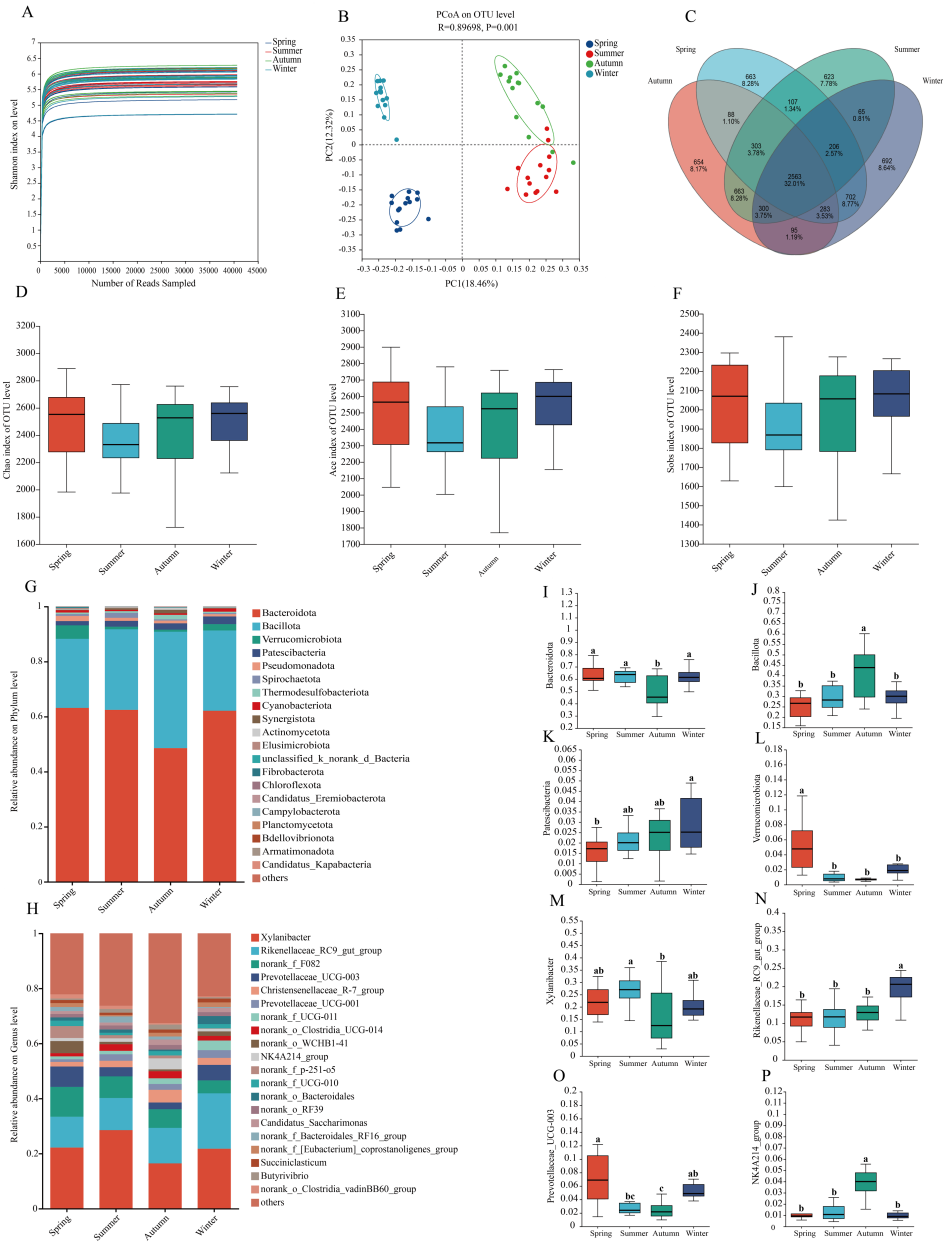
Diversity and richness indicators and OTUs of ruminal bacteria and classification of the bacterial community composition among different seasons. **(A)** Dilution curves across different seasons. **(B)** Principal coordinate analysis (PCoA) of rumen microbial communities. **(C)** A Venn diagram showing the different and similar OTUs among different seasons. **(D)** The Chao index of different seasons. **(E)** The Ace index of different seasons. **(F)** The Sobs index of different seasons. **(G)** Relative abundance of bacterial community at the phylum level. **(H)** Relative abundance of bacterial community at the genus level. **(I)** *Bacteroidota*. **(J)** *Bacillota*. **(K)** *Patescibacteria*. **(L)** *Verrucomicrobiota*. **(M)** *Xylanibacter*. **(N)** *Rikenellaceae_RC9_gut_group*. **(O)** *Prevotellaceae_UCG-003*. **(P)** *NK4A214_group*. The same letter or no letter indicates no significant difference (*p* > 0.05), while different letters indicate a significant difference (*p* < 0.05). The same applies to the figure below.

The rumen microbiota of Altay sheep across different grazing seasons is shown in Figure 4. As illustrated in Figure 4A, the species accumulation curves approached a plateau with increasing sample size, indicating that the sample size was sufficient to estimate community richness. Analysis of Similarities (ANOSIM) was performed to test the differences between groups. The ANOSIM results (*R* = 0.89698 > 0, *p* = 0.001; Figure 4B) revealed significant differences in bacterial community structure among seasons. Based on 97% species similarity, a total of 2,563 operational taxonomic units (OTUs) were common to all samples. Among these, 663 OTUs were unique to spring, 623 to summer, 654 to autumn, and 692 to winter. No significant differences were observed in the Chao1, Ace, and Sobs indices of rumen bacteria in Altay sheep across different grazing seasons (*p* > 0.05), indicating that season had no significant effect on rumen microbial richness. At the phylum level, the predominant bacterial phyla with relatively high abundance across all groups were Bacteroidota, Bacillota, Verrucomicrobiota, Patescibacteria, and Pseudomonadota. At the genus level, the top five most abundant genera in all groups were *Xylanibacter*, *Rikenellaceae_RC9_gut_group*, *norank_f_F082*, *Prevotellaceae_UCG-003*, and *Christensenellaceae_R-7_group*. Differential analysis revealed significant variations in microbial abundance at the genus level. The relative abundance of *Xylanibacter* in summer was significantly higher than that in autumn (*p* < 0.05), while *Rikenellaceae_RC9_gut_group* exhibited its highest relative abundance in winter (*p* < 0.05). The relative abundance of *Prevotellaceae_UCG-003* in spring was significantly greater than that in other seasons (*p* < 0.05), whereas *NK4A214*_group demonstrated significantly higher relative abundance in autumn compared to other seasons (*p* < 0.05). At the phylum level, the relative abundance of Bacteroidota in autumn was significantly lower than that in other seasons (*p* < 0.05), while the relative abundance of Bacillota increased significantly (*p* < 0.05). The relative abundance of Patescibacteria in winter was significantly higher than that in spring (*p* < 0.05). Additionally, the relative abundance of Verrucomicrobiota in spring was significantly elevated compared to other seasons (*p* < 0.05).

**Figure 5:**
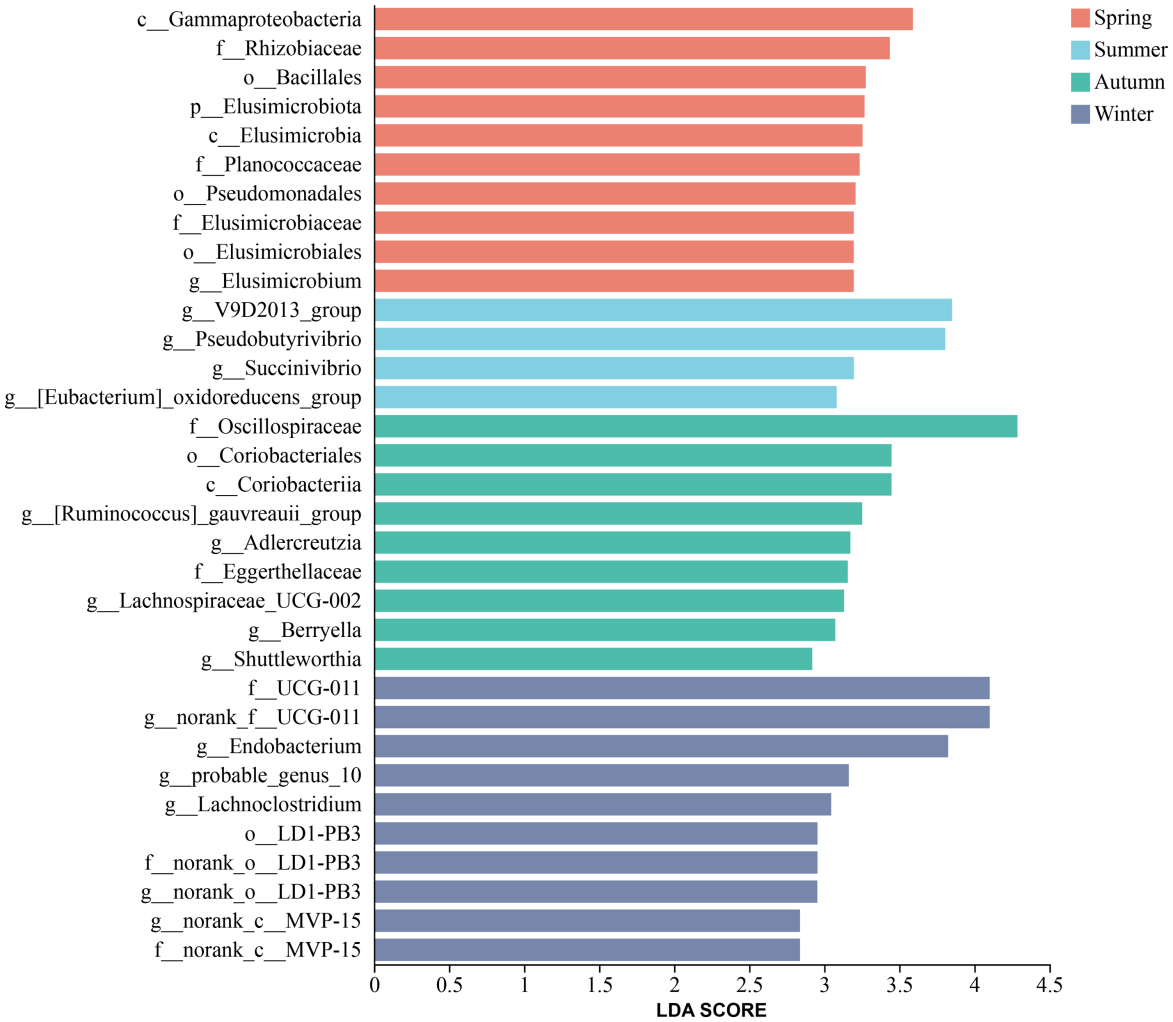
Bacterial biomarkers in the rumen content across different seasons (LDA > 2.5).

LEfSe analysis (Figure 5) was used to analyze the microbial community biomarkers of Altay sheep grazing in different seasons. The results showed that *g_Elusimicrobium* and *g_Pseudomonas* were significantly enriched in the rumen of Altay sheep in spring; *g_V9D2013_group*, *g_Pseudobutyrivibrio*, *g_Succinivibrio*, and *g_[Eubacterium]_oxidoreducens_group* were significantly enriched in the summer; In autumn, *g_[Ruminococcus]_gauvreauii_group*, *g_Lachnospiraceae_UCG-002*, *g_Adlercreutzia*, *g_Berryella*, *g_Solobacterium*, and *g__Shuttleworthia* showed higher enrichment significance; *g_Endobacterium* and *g_probable_genus_10* exhibited higher enrichment significance in winter.

## 4 Discussion

### 4.1 The effect of grazing season on average daily weight gain, feed intake, and apparent nutrient digestibility in Altay sheep

Average daily gain (ADG), feed intake, and nutrient digestibility serve as key indicators for evaluating growth performance and feed efficiency in ruminants. These three parameters are interconnected and collectively influence the overall effectiveness of a feeding regimen. As demonstrated by Luo et al. (2022), diets deficient in either energy or protein can suppress the growth and development of sheep. Consistent with this, our results indicate that summer forage, which was characterized by higher crude protein and lower fiber content, demonstrated enhanced palatability. Consequently, the digestibility of CP, EE, NDF, ADF, and DM in Altay sheep was significantly improved, resulting in a markedly higher ADG compared to other seasons. This observation aligns with previous findings. Furthermore, the feed intake of Altay sheep followed a pattern of initial increase followed by a decrease across the seasonal progression. It is noteworthy that the dry matter intake (DMI) in autumn was significantly higher than that in summer, whereas the average daily gain (ADG) was lower. We hypothesize that autumn forage, being rich in protein and energy, provides high-quality nutrition that facilitates body condition recovery and fat deposition, thereby allowing the animals to establish sufficient physiological reserves for the impending cold season. Moreover, despite its high protein content, the elevated fiber content in autumn forage relative to summer likely reduces overall nutrient digestibility, which may necessitate increased intake to meet nutritional requirements. The high moisture content and consequent lower dry matter content of summer forage may promote satiety in grazing sheep, potentially explaining the lower DMI observed in summer compared to autumn. Our study revealed that Altay sheep experienced weight stasis during winter, which is likely attributable to the low crude protein content, high fiber content, and overall poor forage quality characteristic of this season. These factors collectively led to reduced digestibility, resulting in an inadequate nutrient supply from forage that failed to meet the sheep’s growth requirements (Bourdon et al., 1999; Zhang et al., 2020). Additionally, previous studies report that consumption of cold water by goats during winter can reduce the apparent digestibility of nutrients including CP, EE, NDF, and ADF (Guo T. et al., 2024), a factor that may have also contributed to the reduced ADG observed in Altay sheep during winter.

### 4.2 The effect of grazing season on blood biochemical indicators of Altay sheep

Blood metabolites and hormones represent critical biomarkers in ruminants, providing insights into physiological homeostasis, nutrient partitioning, and health status. The elevated albumin (ALB) and total protein (TP) levels during summer and autumn indicate adequate protein nutrition and enhanced antioxidant capacity, consistent with the high-quality forage available in these seasons (Dikhanbayeva et al., 2021). AST and ALT are key enzymes in amino acid metabolism, catalyzing the transamination of amino acids to keto acids. If liver tissue cells are damaged, leading to lesions and impaired function, AST and ALT in the body enter the bloodstream, increasing their activity (Li et al., 2024). The rise in AST and ALT activities in summer and autumn suggests an increased hepatic metabolic burden, likely due to the higher nutrient load from abundant forage. The seasonal pattern of blood urea nitrogen (BUN) closely mirrored the crude protein content of the pasture, being highest in spring and autumn, which reflects increased dietary protein intake and ruminal ammonia absorption (Bai et al., 2015). LD is a key enzyme in glycolysis and plays a core role in energy metabolism. Its activity is positively correlated with dietary energy levels (Fresco et al., 2010). This study found that lactate dehydrogenase levels in the blood of Altay sheep were significantly lower in winter compared to spring and autumn, consistent with previous research findings. In autumn, GH levels decrease to promote fat accumulation, preparing for the short photoperiod. IGF-1 critically modulates brown adipose tissue development in adipocytes, potentiating adaptive non-shivering thermogenesis capacity (Zhang et al., 2022). Concurrently, the lower T3 and T4 levels in summer suggest a metabolic down-regulation to mitigate heat stress, whereas their recovery in winter is crucial for sustaining basal metabolic rate under cold conditions (Zhang et al., 2022).

### 4.3 The effect of grazing season on the antioxidant capacity of Altay sheep

During animal growth and metabolism, free radicals are continuously generated. Under healthy physiological conditions, the intrinsic redox system of the animal body maintains relative stability by perpetually producing antioxidants through metabolic processes to neutralize the continuously generated free radicals, thereby sustaining a homeostatic equilibrium within the organism (Fu et al., 2021). Serum SOD, GSH-Px, and CAT are key indicators of an animal’s antioxidant capacity, while serum MDA concentration serves as a reliable marker of oxidative damage in animals (Hao et al., 2021). The findings of this study demonstrated an increased MDA concentration in summer. During the hot season, the activities of key antioxidant enzymes were compromised, as indicated by significantly reduced SOD and CAT activities. Although the change in GSH-Px activity was not statistically significant, a decreasing trend was observed in summer. These findings are largely congruent with previous studies. The study by Guo et al. (2018) confirmed that heat stress led to reduced SOD and GSH-Px activities along with elevated MDA levels in lactating dairy cows. High-temperature environments increase lipid peroxidation and inhibit antioxidant enzyme activity (Liu et al., 2014). Sahin and Gümüşlü (2004) research demonstrated significant elevations in SOD and GSH-Px activities within murine cerebral and hepatic tissues following cold exposure. In alignment with these reports, we observed an increasing trend in serum SOD and GSH-Px activities during winter. Collectively, these results suggest that the antioxidant defense system in grazing sheep was impaired during the hot season, supporting the widely held view that sheep possess greater tolerance to cold stress than to heat stress.

### 4.4 The effect of grazing season on inflammatory factors and immune performance in Altay sheep

Immunoglobulins IgA, IgG, and IgM are specific glycoproteins produced by B lymphocytes, serving as the primary antibodies mediators of humoral immunity and representing crucial indicators of immune competence. Serum cytokines play pivotal roles in orchestrating immune responses and serve as sensitive indicators of the health status in Altay sheep. When exposed to exogenous stimuli or injuries, the body may develop inflammation, accompanied by the production of inflammatory factors and the recruitment and activation of immune cells (Wiering and Tacke, 2023). Based on their primary functions, cytokines are broadly classified into pro-inflammatory and anti-inflammatory subtypes. The concentrations of circulating immunoglobulins are known to be modulated by multiple factors, including the intensity and duration of heat stress, animal breed, and physiological status. Heat stress activates both peripheral and central receptors, leading to stimulation of the hypothalamic–pituitary–adrenal (HPA) axis and subsequent increased glucocorticoid secretion. This process subsequently suppresses lymphocyte proliferation and differentiation, ultimately impairing cellular and humoral immune responses and compromising overall immune function (Chauhan et al., 2021). The literature reports that under heat stress conditions, the IgA, IgM, and IgG content in cow’s milk serum is significantly reduced (Gupta et al., 2022). The present study demonstrated that serum immunoglobulin concentrations in Altay sheep during summer were significantly lower than those in spring and winter, which aligns with the aforementioned findings. During stress, limited secretion of pro-inflammatory cytokines such as TNF-α and IL-6 exerts protective effects on the organism, whereas their overproduction can induce tissue and organ damage, thereby compromising the immune system (Kandasamy et al., 2011). Furthermore, elevated levels of IL-1β and IL-6 have been documented in various animal models following cold stress (Zhang et al., 2015). When exposed to temperatures 3 °C below the normal rearing temperature and cold stimulation, the expression levels of IL-6 and IFN-γ in broiler chickens increased significantly, while TNF-α levels were higher than those under normal rearing conditions (Xu et al., 2019). In this study, cytokine levels were significantly higher in spring and winter than in summer, consistent with the aforementioned results. In summary, environmental temperature stress appears to be a primary driver of the significant seasonal fluctuations observed in immune parameters of grazing sheep.

### 4.5 The effect of grazing season on rumen fermentation parameters in Altay sheep

Rumen fluid pH serves as a key parameter reflecting fermentation conditions in the rumen, and its relative stability is crucial for microbial viability and enzymatic function. The present study found that the rumen pH of Altay sheep during summer was significantly lower than in other seasons, potentially attributable to the reduced NDF content in summer forage, where extensive carbohydrate fermentation decreased the ruminal pH (Li et al., 2024). The NH_3_-N content in rumen fluid reflects the efficiency of rumen microorganisms in utilizing feed protein and non-protein nitrogen, and its level is affected by factors such as protein intake and protein degradation rate. Ruminal NH₃-N concentration is positively correlated with the dietary protein content (Moss et al., 2019). The lower crude protein content of pasture in spring and winter led to reduced protein intake by Altay sheep, which corresponded to significantly lower ruminal NH₃-N concentrations—a finding consistent with previous reports. The concentration of volatile fatty acids (VFAs) produced during rumen fermentation is a key indicator of fermentative activity. VFAs are mainly absorbed across the rumen epithelium and serve crucial roles in supporting immune function and growth in ruminants (Cone and Becker, 2012). Acetic acid, propionic acid, and butyric acid are the main components of volatile fatty acids in the rumen, and their levels vary with changes in forage nutrient content, accounting for over 95% of total volatile fatty acids (Nolan et al., 2014). In the present study, the concentrations of acetate, propionate, and total volatile fatty acids in the rumen of grazing Altay sheep during spring and summer were significantly higher than those in autumn and winter. This pattern is likely due to the abundant forage availability and superior nutritional quality in spring and summer, which supports higher voluntary intake by grazing sheep. The provision of sufficient fermentable substrates to the rumen microbiota thus promoted enhanced VFA production (Giger-Reverdin et al., 2014). Research indicates that the A:P ratio in grazing yaks is significantly lower during the warm seasons compared to the cold seasons (He et al., 2024). This finding aligns with the fact that pasture grasses during the warm season contain higher levels of fermentable carbohydrates and lower fiber content, which promotes the proliferation of propionic acid-producing bacteria, thereby enhancing propionic acid production. A lower A:P ratio indicates a higher efficiency in converting dietary energy into metabolizable glucose, supporting the higher average daily gain observed in summer and the fat deposition process in autumn. On the contrary, the significantly increased A:P ratio in winter reflects a typical fiber decomposition fermentation pattern (Yang et al., 2022). Branched-chain volatile fatty acids (BCVFAs), primarily isobutyrate and isovalerate, are produced in the rumen. BCVFAs serve as essential growth factors for many fibrolytic rumen bacteria (Getachew et al., 2004). In this study, the ruminal concentrations of isovalerate and isobutyrate in grazing sheep during spring and summer were significantly higher than those in winter, which may be attributed to the more readily degradable carbohydrates in spring and summer forages. In summary, seasonal variations in the nutritional composition of forages are directly reflected in corresponding shifts in rumen fermentation parameters.

### 4.6 The effect of grazing season on the rumen microbiota of Altay sheep

The rumen microbiota performs critical functions in ruminants, encompassing feed fermentation, immune regulation, and energy supply, while also exerting a significant influence on host phenotypes. The present study observed no significant differences in the alpha-diversity indices of rumen bacterial com munities in Altay sheep across different grazing seasons, indicating that both the diversity and richness of rumen microbiota remained relatively stable to adapt to seasonal variations in forage nutrition. Previous studies have shown that an increase in the relative abundance of the Bacillota phylum and a decrease in the relative abundance of the Bacteroidetes phylum can promote goat growth (Min et al., 2019). Our results revealed a lower relative abundance of Bacteroidota and a concurrent increase in Bacillota during autumn. This suggests that during autumn, when sheep consume high-quality forage, the enrichment of Bacillota may enhance nutrient acquisition, thereby promoting growth performance and fat deposition. Given the prolonged cold season and poor forage quality in the Altay region, the enrichment of Bacteroidota in the rumen during spring and winter likely facilitates optimal nutrient utilization to meet survival requirements (Do et al., 2018). Furthermore, the significant enrichment of Patescibacteria in winter suggests a community-level adaptation to enhance VFA utilization efficiency under energy-limiting conditions (Du et al., 2024). *Rikenellaceae_RC9_gut_group* and *Prevotellaceae_UCG-003* can effectively degrade insoluble cellulose in feed during winter enrichment, and are positively correlated with the production of acetate, maximizing the extraction of energy from low-quality winter forage to cope with the cold winter environment (Guo Y. et al., 2024; Wang D. et al., 2023). Furthermore, the enrichment of *Xylanibacter* during summer likely facilitates the rapid degradation of high-protein forages, thereby enhancing energy turnover and subsequently supporting immune function. In summary, the composition and function of the rumen microbiota adapt to seasonal variations in forage nutrition during grazing, thereby fulfilling the host’s physiological requirements. While this study focuses on the adaptation to seasonal forage nutrient dynamics, it is crucial to acknowledge that these fluctuations are inextricably linked with significant changes in environmental temperature, which itself is a potent modulator of physiology, immunity, and rumen function. For instance, the suppressed antioxidant capacity and immunoglobulin levels (IgA, IgG, IgM) during summer are classic hallmarks of heat stress, which can directly impair immune function and increase oxidative damage, likely compounding any subtle effects of dietary change. Conversely, the weight stasis and metabolic adjustments in winter occur not only due to poor forage quality but also in response to the high energy demands of cold stress. Therefore, the outstanding adaptability of Altay sheep should be regarded as a comprehensive response to nutritional deficiency and environmental temperature. The rumen microbiota, as a key interface, helps to buffer these environmental pressures.

## 5 Conclusion

During summer and autumn, the abundant nutrient content in forages facilitated high feed intake in Altay sheep, which promoted body weight gain through enhanced rumen fermentation efficiency and nutrient digestibility. In contrast, the inferior nutritional quality of winter forages led to reduced feed intake, resulting in lower apparent nutrient digestibility and compromised growth performance. Furthermore, significant seasonal variations were observed in blood biochemical parameters, immune function, and antioxidant capacity. Specifically, the relative abundances of Bacillota and *NK4A214_group* were significantly increased in autumn, facilitating enhanced nutrient acquisition and fat deposition. In winter, the relative abundance of *Rikenellaceae_RC9_gut_group* and *Prevotellaceae_UCG-003* is relatively high, which is conducive to fiber degradation. Meanwhile, these microbial adaptations enable animals to extract essential volatile fatty acids from low-quality feed, thereby helping them cope with the shortage of nutritional resources. Meet the survival needs of this difficult season. In conclusion, the blood characteristics and rumen microbiota of Altay sheep exhibited significant plasticity in response to seasonal grazing patterns. These adaptive changes enable the animals to effectively counteract seasonal fluctuations in forage nutrients availability and external environmental conditions, thereby fulfilling their survival requirements.

## Data Availability

The data presented in this study are deposited in the NCBI BioProject repository under accession number PRJNA1308996.
